# Clinical presentation and aetiologies of acute or complicated headache among HIV-seropositive patients in a Ugandan clinic

**DOI:** 10.1186/1758-2652-12-21

**Published:** 2009-09-19

**Authors:** Michael Katwere, Andrew Kambugu, Theresa Piloya, Matthew Wong, Brett Hendel-Paterson, Merle A Sande, Allan Ronald, Elly Katabira, Edward M Were, Joris Menten, Robert Colebunders

**Affiliations:** 1 Adult HIV Clinic, Infectious Diseases Institute, Makerere University, Kampala, Uganda; 2 Department of Neurology, University of Virginia, Charlottesville, USA; 3 Department of Medicine, University of Minnesota, USA; 4 Faculty of Medicine, University of Washington, USA; 5 Department of Internal Medicine, University of Manitoba, Canada; 6 Department of Clinical Sciences, Institute of Tropical Medicine, Antwerp, Belgium; 7 Faculty of Medicine, University of Antwerp, Belgium; 8 Uganda AIDS Commission, Kampala, Uganda

## Abstract

**Background:**

We set out to define the relative prevalence and common presentations of the various aetiologies of headache within an ambulant HIV-seropositive adult population in Kampala, Uganda.

**Methods:**

We conducted a prospective study of adult HIV-1-seropositive ambulatory patients consecutively presenting with new onset headaches. Patients were classified as focal-febrile, focal-afebrile, non-focal-febrile or non-focal-afebrile, depending on presence or absence of fever and localizing neurological signs. Further management followed along a pre-defined diagnostic algorithm to an endpoint of a diagnosis. We assessed outcomes during four months of follow up.

**Results:**

One hundred and eighty patients were enrolled (72% women). Most subjects presented at WHO clinical stages III and IV of HIV disease, with a median Karnofsky performance rating of 70% (IQR 60-80).

The most common diagnoses were cryptococcal meningitis (28%, n = 50) and bacterial sinusitis (31%, n = 56). Less frequent diagnoses included cerebral toxoplasmosis (4%, n = 7), and tuberculous meningitis (4%, n = 7). Thirty-two (18%) had other diagnoses (malaria, bacteraemia, etc.). No aetiology could be elucidated in 28 persons (15%). Overall mortality was 13.3% (24 of 180) after four months of follow up. Those without an established headache aetiology had good clinical outcomes, with only one death (4% mortality), and 86% were ambulatory at four months.

**Conclusion:**

In an African HIV-infected ambulatory population presenting with new onset headache, aetiology was found in at least 70%. Cryptococcal meningitis and sinusitis accounted for more than half of the cases.

## Background

In the industrialized world, headache is a common complaint amongst both HIV-negative and HIV-positive individuals. In HIV-negative patients, the cause of headache is rarely secondary to significant intracranial pathology [1-3], but in HIV-positive patients, the risk of a secondary "serious" cause of headache is much higher, especially in those who are immunocompromised. In this group, the frequency of "serious" aetiologies depends on the clinical setting, with frequencies ranging from 4% to 82% [4,5].

The primary diagnostic procedure for headache in HIV-positive subjects is neuroimaging [6], with some experts recommending computerized tomography (CT) or magnetic resonance imaging in all HIV-positive patients with headache [7]. This presents a unique challenge to the care of HIV-positive patients in sub-Saharan Africa, where access to diagnostic neuroradiologic expertise and equipment is severely limited [8,9].

A study from South Africa noted that most patients with advanced AIDS complained of pain, and 42% of these had headache as a major pain site [10]. To our knowledge, no studies to date have been done to estimate the relative frequencies of the various aetiologies of headache in an HIV population in sub-Saharan Africa.

The purpose of this study was to determine these relative frequencies and their associated clinical presentations in an ambulatory HIV-positive population in Kampala, Uganda.

In particular we wanted to determine if there were elements in the clinical presentation that could allow a clinician to differentiate a headache secondary to a "serious" cause from one secondary to a "benign" cause. The identification of such elements may allow more efficient use of constrained resources, such as neuro-imaging and other expensive diagnostic tests.

## Methods

The study was conducted in the Adult Infectious Diseases Clinic (Adult IDC) in Kampala, Uganda. The Adult IDC is a specialized semi-autonomous section of the Outpatients' Department of the Mulago National Referral Hospital. It is located in an urban setting, with a catchment area of at least three million people in and around the city of Kampala. It is a referral centre for HIV-infected adults at primary, secondary and tertiary levels of care. The clinic receives referrals from HIV/AIDS care centres situated outside Kampala in all the five regions of Uganda.

As of March 2004, the clinic had about 10,000 HIV-infected adults in care (about 65% of them being female). More than 50% of the patients were at WHO HIV clinical stages III and IV at the time of recruitment into care, with an antiretroviral therapy (ART) coverage of about 30%. Patients who need in-patient clinical care are referred to the Mulago Hospital in-patient medical wards upon assessment and initial resuscitation within the clinic.

The study was performed during a 12-month period from March 2004 to February 2005. All patients with headache as one of their main complaints during the study period were consecutively referred to one of two study physicians.

In order to be included in the study, subjects had to have a positive ELISA HIV-1 test and a positive confirmatory Western blot HIV-1 test. Individuals were excluded if they were younger than 18 years of age.

They were eligible for the study if one of their main complaints was a headache, and if it was: their first headache; different in character from previous headaches; the worst headache they ever experienced; or a persistent headache (more than 72 hours) despite using measures that previously relieved their headache.

In addition, they were included in the study if their headache was accompanied by fever (axillary temperature greater than 37.5 degrees Celsius), vomiting, new or increased frequency of seizures, altered mental state, neck stiffness, or any new focal neurologic symptom or sign.

Medical officers collected information on demographics, history of the present illness, neurologic symptoms, past medical and medication history, and functional status. Patients were asked to score the severity of their headache using a scale from 1 to 10. In addition, all study participants had a general medical and neurologic examination. All this information was recorded on standardized case report forms.

The aetiology of each subjects' headache was diagnosed using standardized criteria established before the study commenced; these are listed below (Table 1). Lumbar puncture and cerebrospinal fluid examination were performed for all enrolled subjects who did not have a focal neurological deficit and who did not meet the case definition of bacterial sinusitis (see Table 1).

**Table 1:** Diagnostic criteria utilised in the study

*Diagnosis*	*Case definition*
**Cryptococcal meningitis**	Presence of Cryptococcus in the cerebrospinal fluid (CSF) by India ink examination, CSF fungal culture, or positive serum cryptococcal antigen (CRAG) test.
	
**Cerebral toxoplasmosis**	Headache accompanied by a focal neurological deficit, with clinical improvement on empiric cotrimoxazole therapy within 14 days of initiation. A positive CT of the brain revealing characteristic ring-enhancing lesions was not required.
	
**Bacterial sinusitis**	Clinical symptoms and signs (rhinorrhoea, nasal stuffiness, headache worse when bending over, frontal or maxillary sinus pain, and tenderness on percussion), with or without air fluid levels on skull film, and response to antibiotic treatment.
	
**Tuberculous meningitis**	Mycobacterium tuberculosis demonstrated in CSF by Ziehl-Neelsen staining and/or mycobacterial culture (Loewenstein-Jensen culture medium); or mycobacterium tuberculosis not demonstrated in the CSF, but: (A) CSF findings compatible with CSF protein >60 g/dL, and >200 cells/mm^3 ^with lymphocytic predominance; (B) evidence of extra central nervous system tuberculosis; (C) exclusion of other aetiologies of meningitis; and (D) positive response to anti-tuberculous therapy
	
**Viral meningitis**	On the basis of mild-moderate CSF pleocytosis (<100 leukocytes/ml) and moderately elevated protein in CSF (40-150 g/dL) with negative CSF fungal/bacterial cultures, negative Ziehl-Neelsen and gram stains of CSF, negative serum CRAG and exclusion of tuberculosis at other sites.

**Patients' functional status **was rated using the Karnofsky Performance scale shown here:

**Percent (%)**   Description

**100**   Normal; no complaints; no evidence of disease.

**90**   Able to carry on normal activity; minor signs or symptoms of disease.

**80**   Normal activity with effort; some signs or symptoms of disease.

**70**   Cares for self; unable to carry on normal activity or to do active work.

**60**   Requires occasional assistance, but is able to care for most of one's needs.

**50**   Requires considerable assistance and frequent medical care.

**40**   Disabled; requires special care and assistance.

**30**   Severely disabled; hospitalization indicated although death not imminent.

**20**   Very sick; hospitalization necessary; active, supportive treatment necessary.

**10**   Moribund, fatal processes progressing rapidly.

**0**   Dead

Patients were classified as focal-febrile, focal-afebrile, non-focal-febrile or non-focal-afebrile depending on presence or absence of localizing neurological signs and presence or absence of pyrexia. Further workup followed along a predefined study workup plan, to an endpoint of a diagnosis.

CT scanning was only performed in the following scenarios: (A) patients with a focal neurological deficit that did not improve within 10 days of empiric toxoplasmosis therapy; (B) patients with a persistent headache after a standardised workup and treatment; or (C) patients whose comprehensive diagnostic workup did not reveal a diagnosis for their headache.

Patients received appropriate clinical management, and were followed by the study physicians until four months post-diagnosis or death. The patients' investigations and management were paid for by the study and by the Adult IDC. At the start of the study, patients had to pay for their ART themselves, but from July 2004, donor programmes began to provide access for many of the study participants.

Prevalences of the aetiologies of headaches were estimated together with 95% confidence intervals, calculated using Wilson's score method. The association between possible predictors and each of the most common diagnoses was assessed using Fisher's Exact test. Predictors associated with a "serious diagnosis" (defined as cryptococcal meningitis, tuberculous meningitis or cerebral toxoplasmosis) were assessed using multiple logistic regression models.

Variables significantly associated (p-value ≤ 0.050) were entered in a logistic regression model followed by backward elimination removing variables from the model at 5% significance using likelihood ratio tests. All statistical analyses were performed using SAS 9.1 (SAS Institute Inc., Cary, NC, USA) and R 2.6 (R Foundation for Statistical Computing, Vienna, Austria).

The study was approved by the local research ethics committee and by the Uganda National Council for Science and Technology. Written informed consent was obtained from all individuals in the study.

## Results

During the study period (March 2004 to February 2005), 273 persons presented with headaches and were referred for study screening. We excluded 86 subjects (31.5%) who did not meet the study inclusion criteria (one was HIV seronegative on confirmatory testing; 85 presented with headaches that did not meet the enrolment criteria described in the Methods section). Seven patients meeting study criteria declined consent mainly due to personal and cultural fears regarding the use of lumbar puncture as a potential investigation tool. Finally, 180 subjects, who met the study eligibility criteria, were enrolled.

Patients' characteristics at the time of enrolment are presented in Table 2. Women accounted for 72% of the enrolled subjects, which is consistent with the demographics of the clinic. A generalized or frontal headache was reported by 78% of the study participants with a median severity score of eight. The majority presented with advanced HIV, with 78% at WHO HIV clinical stages III or IV. The median Karnofsky performance rating was 70 (IQR 60-80).

**Table 2:** Patients' characteristics at study enrolment

Patient characteristics	n (%)
Total N	180
Sex^†^	
Men: n (%)	51 (28)
Women: n (%)	128 (72)
Age: median (IQR)	35 (30-41)
Location^†^	
Frontal: n (%)	68 (39)
Temporal: n (%)	27 (15)
Occipital: n (%)	9 (5)
Vertex: n (%)	2 (1)
Generalized: n (%)	69 (39)
Headache score^†^: median (IQR)	8 (6-10)
Headache duration (days)^†^: median (IQR)	10 (5-21)
WHO stage^†^:	
I or II: n (%)	38 (21)
III: n (%)	73 (41)
IV: n (%)	66 (37)
Patient on ART: n (%)	53 (29)
Karnofsky performance score^†^: median (IQR)	70 (60-80)
CD4 count^†^: median (IQR)	108 (20-239)
Workup classification^†^:	
Focal/febrile	3 (2)
Non-focal/febrile	19 (11)
Focal/afebrile	11 (6)
Non-focal/afebrile	144 (81)

^† ^Number of patients with data missing: gender (1), location (5), headache score (6), headache duration (1), WHO stage (3), Karnofsky performance score (4), CD4 (10), workup classification (3).

Less than 20% of the study subjects presented with either fever or focal neurological signs (Table 2).

Almost 60% of the headache presentations were attributable either to Cryptococcus neoformans meningitis or to presumed bacterial sinusitis (Table 3). The clinical features of the main aetiological diagnoses are shown in Table 4. Thirty two (18%) subjects presented with features of meningeal irritation and/or raised intracranial pressure. Twenty-five (78%) of these 32 patients were diagnosed with cryptococcal meningitis. Cryptococcal meningitis was diagnosed in 10 (77%) of 13 patients with neck stiffness; in six (67%) of nine patients with a positive Kernig's sign and in seven (70%) of 10 patients with papilloedema at baseline. Two subjects subsequently diagnosed with cryptococcal meningitis did not present with features of meningeal irritation at enrolment.

**Table 3:** Number and percentage of patients by headache aetiology

Diagnosis	N	Percentage (95% confidence interval)
Cryptococcal meningitis	50	28 (22-35)
Bacterial sinusitis	56	31 (24-38)
Cerebral toxoplasmosis	7	4 (2-8)
Tuberculous (TB) meningitis	7	4 (2-8)
Other:	32	18 (13-24)
*Viral meningitis*	6	3 (2-7)
*Malaria*	5	3 (1--6)
*TB adenitis/abdominal TB*	3	2 (1-5)
*Depression/anxiety*	2	1 (0-4)
*CMV retinitis*	2	1 (0-4)
*Drug-induced headache*	2	1 (0-4)
*Partially treated bacterial meningitis*	1	1 (0-3)
*Otitis media*	1	1 (0-3)
*Tonsillitis*	1	1 (0-3)
*Aphthous ulcer*	1	1 (0-3)
*Central retinal vein occlusion*	1	1 (0-3)
*HIV associated nephropathy*	1	1 (0-3)
*Urinary tract infection*	1	1 (0-3)
*Herpes zoster ophthalmicus*	1	1 (0-3)
*Bell's palsy*	1	1 (0-3)
*Staphylococcus aureus bacteraemia*	1	1 (0-3)
*Cerebro-vascular accident*	1	1 (0-3)
*Hepatitis*	1	1 (0-3)
Unknown	28	16 (11-22)

**Table 4:** Clinical features associated with the main diagnoses (number of patients by characteristic and main diagnosis)

	CM (N = 50)	Sinusitis (N = 56)	Toxoplasmosis (N = 7)	TBM (N = 7)	Other (N = 32)	Unknown (N = 28)
***Gender***						
**Male**	20	4	4	3	8	12
**Female**	29	52	3	4	24	16
p-value	0.040	< 0.001	0.103	0.408	0.829	0.073
***Location of headache*:**						
**Frontal**	13	34	1	5	6	9
**Temporal**	4	5	2	1	8	7
**Occipital**	5	2	0	0	2	0
**Vertex**	0	2	0	0	0	0
**Generalised**	27	12	4	1	15	10
p-value	0.008	< 0.001	0.461	0.392	0.077	0.400
***Headache duration:***						
**0-13 days**	25	32	4	4	14	13
**14-27 days**	12	9	1	1	15	7
**>28 days**	12	15	2	2	3	8
p-value	1.000	0.163	0.888	0.888	0.006	0.712
***WHO stage*:**						
**I or II**	3	17	0	0	9	9
**III**	16	24	2	4	15	12
**IV**	30	14	5	3	8	6
p-value	< 0.001	0.041	0.129	0.480	0.240	0.133
***CD4 count:***						
**<50**	31	11	1	2	14	8
**50-199**	15	18	3	3	6	6
**≥ 200**	1	24	1	2	11	13
p-value	< 0.001	0.001	0.451	0.801	0.376	0.115
***Workup classification:***						
**Focal/febrile**	1	0	0	0	1	1
**Non-focal/febrile**	5	5	1	0	7	1
**Focal/afebrile**	3	0	4	1	3	0
**Non-focal/afebrile**	39	50	2	6	21	26
p-value	1.000	0.048	< 0.001	0.550	0.040	0.182
***Change in head position worsens headache:***						
**No**	2	12	3	1	9	10
**Yes**	47	42	4	6	22	18
p-value	< 0.001	0.842	0.162	1.000	0.232	0.046
***Meningeal signs and/or raised intracranial pressure:***						
**Neck stiffness**	10	0	0	2	1	0
p-value	< 0.001	0.010	1.000	0.084	0.468	0.222
**Positive Kernig's sign**	6	0	0	1	1	1
p-value	0.015	0.059	1.000	0.311	1.000	1.000
**Papilloedema**	7	1	0	1	0	1
p-value	0.005	0.175	1.000	0.277	0.210	1.000
***New onset headache*:**						
**No**	3	5	1	0	1	0
**Yes**	46	49	6	7	30	26
p-value	1.000	0.288	0.344	1.000	1.000	0.362
***Worst headache in patient's lifetime*:**						
**No**	11	16	1	1	6	8
**Yes**	38	39	6	6	26	20
p-value	0.846	0.345	1.000	1.000	0.501	0.631

See note for Table 2 for numbers of patients with missing data.

P-values from Fisher's Exact test comparing those with and without the diagnosis by possible predictor.

CM: Cryptococcal meningitis; TBM: Tuberculous meningitis.

Of the 50 patients with cryptococcal meningitis, 38 (76%) presented with an initial episode of cryptococcal infection, and 12 (24%) presented with either a relapse of the disease or an immune reconstitution event secondary to antiretroviral treatment.

Thirty six (52%) were managed without hospitalization with oral fluconazole; of these, 12 (33%) died. Of the 14 (28%) who were admitted to hospital for amphotericin B treatment, eight (57%) died of either cryptococcal disease or complications of therapy. Of the 50 patients with cryptococcal meningitis, only 17 (34%) received ART. Fourteen patients with cryptococcal meningitis died before they received ART, and six died after initiating ART.

Fifteen percent of the headaches could not be classified aetiologically. These headaches generally improved on oral analgesics; but recurrent headache of mild to moderate severity during follow up was reported. One patient from this group died from presumed ART-related immune reconstitution syndrome during the four months of follow up. Two patients were lost to follow up, the rest (86%) were ambulant, with a Karnofsky performance status greater than 80% at four months of follow up.

Overall, mortality after four months of follow up was 13.3% (24 of 180). Only six (25%) of the 24 study patients who died were on antiretroviral treatment.

In the univariate analysis, the following variables were significantly associated with a diagnosis of cryptococcal meningitis (Table 4): male gender (p = 0.040), a generalised headache (p = 0.008), WHO stage (p < 0.001), CD4 cell count below 200 cells/ml (p < 0.001), change in head position worsens headache (p < 0.001), neck stiffness (p < 0.001), Kernig's sign (p = 0.015), and papilloedema (p = 0.005).

The same variables, apart from location of headache, were associated with a diagnosis of a "serious condition" (that is, cryptococcal meningitis, tuberculous meningitis or cerebral toxoplasmosis). Multiple logistic regression confirmed male gender, CD4 cell count less than 200 cells/ml, change in head position worsens headache, and neck stiffness as significant independent predictors of a diagnosis of cryptococcal meningitis; and male gender, CD4 cell count less than 200 cells/ml, change in head position worsens headache, neck stiffness, and WHO disease stage as significant independent predictors of diagnosis of a "serious condition" (Table 5).

**Table 5:** Features associated with any serious conditions (cryptococcal meningitis, cerebral toxoplasmosis or tuberculous meningitis)

	Cryptococcal meningitis		Serious condition	
	Odds ratio (95% CI)	p-value	Odds ratio (95% CI)	p-value
Sex (male)	3.4 (1.4, 9.0)	0.011	4.0 (1.6, 10.3)	0.003
CD4				
<50	47.3 (8.6, 901)	< 0.001	9.4 (2.6, 46.4)	0.002
50-199	23.5 (4.1, 454)	0.004	7.5 (2.0, 37.4)	0.006
≥200	1 (Ref.)		1 (Ref.)	
Change in position worsens headache	21.4 (3.9, 407)	0.005	6.3 (1.9, 26.0)	0.005
Neck stiffness	9.2 (1.8, 82.8)	0.019	32.2 (4.2, 719)	0.004
WHO stage				
I & II			1 (Ref.)	
III	?	?	4.2 (1.0, 24.4)	0.066
IV	?	?	6.5 (1.6, 37.6)	0.016

Note: Odds ratios, confidence intervals (95% CI) and p-values calculated from multivariate logistic regression model. All variables included in the final multivariate model are shown. Variables considered in the step-wise procedure were: gender, headache location (Cryptococcal meningitis only), WHO stage, CD4 cell count, change in head position worsens headache, neck stiffness, Kernig's sign and papilloedema.

## Discussion

About 60% of the headache presentations in this series of patients were attributable either to cryptococcal meningitis or to sinusitis. Not unexpectedly, clinically severe headache with CD4 counts of below 200 cells/mm^3 ^was more likely to be due to cryptococcal meningitis; for those with CD4 counts of above 200 cells/mm^3^, severe headache was more likely to be due to sinusitis. The diagnosis of sinusitis carried a good prognosis, usually with rapid improvement with antimicrobial treatment and few relapses despite a study population with advanced HIV disease.

The overwhelming predominance of sinusitis in females is unexplained. Perhaps they were more likely to present to the clinic with less severe headache than men. Sinusitis is a major unappreciated challenge in HIV patients. Its frequency was not anticipated, but it has been noted in other studies [11]. However, we also note that our case definition for bacterial sinusitis does not sufficiently rule out a migrainous headache. Therefore it is possible that some of the cases that were diagnosed and managed as sinusitis were attributable to migraine or migrainous headache.

More than 90% of headaches due to cryptococcal meningitis, tuberculous meningitis and toxoplasmosis were exacerbated by changes in head position. This phenomenon is presumably due to elevations of intracranial pressure that are associated with these conditions.

We noted that headache duration, mode of onset of the headache (insidious or acute), and headache severity did not correlate with a diagnosis of cryptococcal meningitis. Neck stiffness and a CD4 cell count of below 200/mm^3 ^predicted cryptococcal disease. However additional investigations, particularly cerebrospinal fluid analysis and serum CRAG test, are needed for a definitive diagnosis. Eighteen (75%) of the 24 deaths in the study population were attributable to cryptococcal meningitis.

In 15% of the cases, no aetiology was discovered after full diagnostic workup. These patients generally improved on analgesic therapy. There were no clinical parameters from our analysis that could clearly predict an "unknown" aetiology. Fortunately, all of the patients, except one, with an unknown aetiology for their headaches improved with supportive therapy alone.

We conclude from this that once other serious and treatable causes have been ruled out by history and physical exam, laboratory and/or imaging, it is reasonable to treat with analgesics, reassurance and close clinical follow up.

Cerebral toxoplasmosis accounted for just less than 4% of the headache aetiologies in our population. This was observed despite the fact that more than 54% of persons with HIV infection in Uganda were reported to have a positive serology for toxoplasmosis [12].

The low prevalence of cerebral toxoplasmosis in our study population may be due to the fact that most of our HIV-seropositive adults are initiated on co-trimoxazole prophylaxis in accordance with the national guidelines for the management of HIV in Uganda. Second, patients with cerebral toxoplasmosis presenting with focal neurological deficits may be more likely to be seen in an in-patient setting.

In addition, some patients with focal neurological symptoms attributable to cerebral toxoplasmosis may not have presented with headache as a major complaint. Such patients would not have been enrolled in this study due to the inclusion and exclusion criteria for the headache symptom.

It is imperative to note that the diagnosis of cerebral toxoplasmosis was empiric, based on improvement of focal neurological symptoms after at least 10 days of co-trimoxazole therapy. Computerised tomography scanning was not a requirement in the study for the diagnosis of cerebral toxoplasmosis. It is therefore possible that there were alternative aetiologies for some of the subjects diagnosed with cerebral toxoplasmosis.

Tuberculous and viral meningitides together accounted for just less than 8% of the aetiologies of headaches. These conditions, however, may be under-diagnosed given the absence of definitive diagnostic kits (for instance, CSF PCR for Epstein Barr, Herpes simplex, etc.) for the viral meningitides and the very low sensitivity [13] of CSF Ziehl-Neelsen staining for the diagnosis of tuberculous meningitis.

We also noted a low prevalence of headache (of any cause) associated with fever (less than 20% of study subjects). This could be due to the fact that our patients frequently utilise over-the-counter medications like non-steroidal anti-inflammatory drugs and paracetamol to alleviate their HIV-related ailments, like pain.

The study had a number of limitations. It was drawn from a patient population of HIV-infected individuals registered at only one care centre. Also, many patients with very severe headaches, neurological localizing findings or seizures may have presented directly to the casualty department of the hospital rather than to an out-patient setting. It was not financially feasible to do CT scanning for all the patients in the study. Finally, viral meningitides and tuberculous meningitis were diagnosed by an exclusion process rather than by definitive technologies, which were not available.

## Conclusion

We conclude that at least 70% of the aetiologies for new onset and/or severe headache in a large African HIV-infected ambulatory population can be adequately diagnosed in resource-limited settings; in our study, cryptococcal meningitis and sinusitis accounted for more than half of these cases.

At least 80% of the diagnoses in our study were arrived at without needing advanced imaging, which is reassuring with regard to HIV/AIDS management in resource-constrained settings. Finally, headaches of unknown aetiology have a relatively good prognosis with supportive therapy alone, as long as more serious causes of headache have been ruled out.

## Competing interests

Each of the authors, MK, AK, TP, MW, BHP, AR, EK, EMW, JM and RC declare that they have no competing interests with respect to this manuscript.

MAS sadly passed away in 2008; he too did not have any competing interests regarding the publication of this manuscript.

## Authors' contributions

MK participated in study set up and conduct, data collection and analysis, drafting and writing of the manuscript. AK participated in the conception of the study, study set up and data analysis. TP participated in study conduct, data collection, data cleaning and manuscript writing. MW was involved in the conception of the study, data analysis and drafting of the manuscript. BHP participated in the drafting and reviewing of the manuscript. MAS participated in study set up, drafting and reviewing of the manuscript. AR participated in conception of the study, drafting and writing of the manuscript. EK was involved in the conception of the study and drafting of the manuscript. EMW participated in the data analysis, drafting and reviewing of the manuscript. JM participated in the data analysis, reviewing and writing of the manuscript. RC participated in the study conduct, reviewing and writing of the manuscript. All the authors reviewed and approved the manuscript.
